# Mycobacterium tuberculosis Infection After Grafting of Infected Bone: A Case Series of Four Patients

**DOI:** 10.7759/cureus.66603

**Published:** 2024-08-10

**Authors:** Oluwafemi Ajibola, Amy W Wolfe, Juzar Ali

**Affiliations:** 1 Pulmonary and Critical Care Medicine, Louisiana State University Health Sciences Center, New Orleans, USA; 2 Pulmonology and Critical Care Medicine, Louisiana State University Health Sciences Center, New Orleans, USA; 3 Section of Pulmonary and Critical Care Medicine, Louisiana State University School of Medicine, New Orleans, USA

**Keywords:** disseminated tuberculosis, public health, donor procurement standards, bone grafting, spinal tuberculosis

## Abstract

Back pain is one of the commonly reported medical symptoms, and the mainstay of treatment is conservative care and rehabilitation, but in severe cases with nerve compression from herniated discs, spondylolisthesis, fractures, or spinal canal stenosis, surgery can be helpful. The use of donor bone grafting is common but associated with some complications, including infection.

We present a case series of four patients who underwent spinal surgery with allograft bone transplantation and developed *Mycobacterium tuberculosis *(MTB) disease due to infected bone grafts. Each patient required 12 months of therapy for MTB disease and had various complications from the required anti-mycobacterial treatment.

After the first outbreak of MTB infection from donor bone grafting in 2021, the tissue procurement organizations implemented the use of nucleic acid amplification testing for MTB in the bone allografts, but this is not the most sensitive test available. This test did not detect the MTB in the tissue that was implicated in the second outbreak, and cultures for MTB did not become positive until the bone had already been distributed and grafted into 36 patients.

In response to both outbreaks, the American Association of Tissue Banks (AATB) has recently published new guidelines, which include recommended criteria and literature reviews to aid with screening out cases that may have MTB and improving safety measures for recipient patients.

## Introduction

Back pain is one of the commonly reported medical symptoms with multiple etiologies, including degenerative disc disease, herniated or ruptured discs, spondylolisthesis, or spinal stenosis [1]. The mainstay of treatment for back pain is conservative care and rehabilitation, but in severe cases with nerve compression from herniated discs, spondylolisthesis, fractures, or spinal canal stenosis, surgery can be helpful [1,2]. 

The bone is well known for its ability to regenerate completely, and the use of bone grafting helps to facilitate this regeneration. Iliac crest autograft has long been considered the gold standard for bone graft in spine surgery, but the acquisition of the autograft is associated with donor site pain and increased operative time, leading to the use of allogeneic bone graft [3,4]. The use of allograft is not free of complications. Allografting is associated with increased risks of rejection, disease transmission, and slower incorporation into the host bone [3]. 

We present a case series of four patients who underwent spinal surgery in 2023 with allograft bone transplantation from the same donor and developed *Mycobacterium tuberculosis* (MTB) disease due to infected bone grafts.

## Case presentation

Case 1 

**Figure 1:**
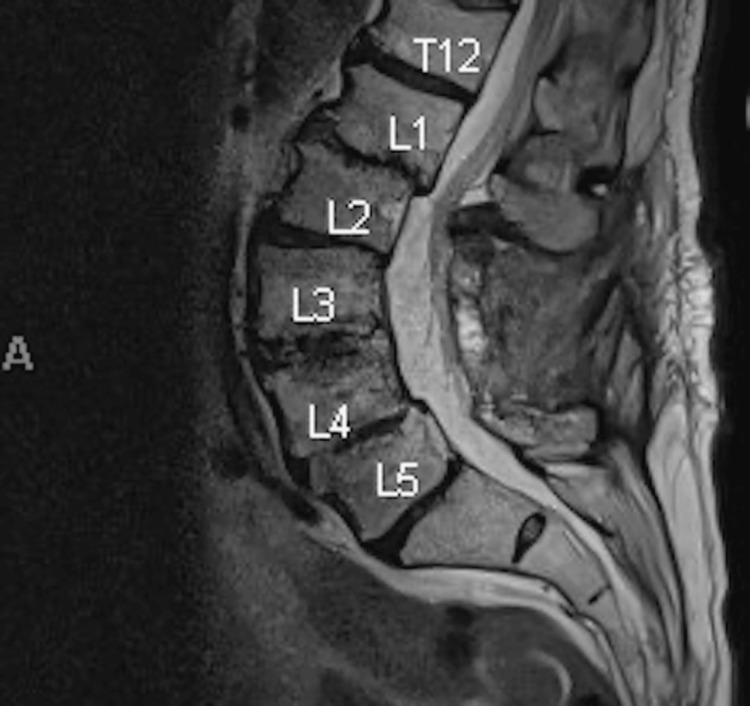
MRI spine of case 1 Soft tissue edema of the laminectomy bed and tiny enhancing area of fluid

A 70-year-old man with a history of hypertension, aortic stenosis with transcatheter aortic valve replacement, and a prior minimal history of one to two years of tobacco use was evaluated for chronic back pain due to spondylolisthesis of the lumbar region and severe spinal stenosis initially requiring lumbar epidural steroid injection. Eventually, he underwent lumbar spinal surgery with allograft transplantation. One month after the surgery, he developed self-limited fever, sore throat, fatigue, headache, and myalgias. Once the national outbreak was identified in July 2023, he was told he had received a bone graft contaminated with MTB. Interferon-gamma release assay (IGRA) testing was positive. Testing for sputum acid fast bacilli (AFB), culture, and chest imaging were all negative. The three-month post-operative magnetic resonance imaging (MRI) of the spine showed soft tissue edema at the laminectomy bed with tiny postsurgical peripherally enhancing fluid collection (Figure 1).

The patient was started on rifampin, isoniazid, pyrazinamide, and ethambutol therapy (RIPE) with vitamin B6 for MTB disease. The treatment course of the patient was complicated by rash and hyperuricemia that required holding medications for a week, followed by modification of his treatment regimen to rifampin, isoniazid, B6 and moxifloxacin as per CDC recommendations. He was subsequently able to tolerate this regimen well. 

Case 2 

A 73-year-old woman with a history of heart failure with preserved ejection fraction, aortic stenosis, transcatheter aortic valve replacement, coronary artery disease, sick sinus syndrome, dual-chamber pacemaker placement, bilateral knee replacement, hypertension, diabetes, chronic kidney disease, paroxysmal atrial fibrillation, and hyperlipidemia was evaluated for chronic back pain due to spondylolisthesis of lumbar region and spinal stenosis. She underwent lumbar spine surgery with allograft transplantation in 2023. One month later, the patient developed recurrent lower back pain, intermittent low-grade fever, malaise, and anorexia. The C-reactive protein (CRP) and erythrocyte sedimentation rate (ESR) were elevated. The computed tomography (CT) scan of the lumbar spine showed potential concern for L5 diskitis or osteomyelitis. She underwent bilateral lumbar incision, drainage, and debridement of the spinal area of concern. Cultures and smears were subsequently negative for all bacteria, AFB, and MTB-PCR. As she had been recently informed that she received a bone graft contaminated with MTB, she was started on RIPE/B6 therapy for clinical diagnosis of spinal MTB infection. IGRA was positive. Her treatment course with RIPE therapy was complicated by hyperuricemia and symptoms of gout that required modification of therapy to rifampin, isoniazid, moxifloxacin, and pyridoxine. She continued to have intermittent symptoms of gout, tendonitis, fatigue, and anemia throughout therapy. 

Case 3 

**Figure 2:**
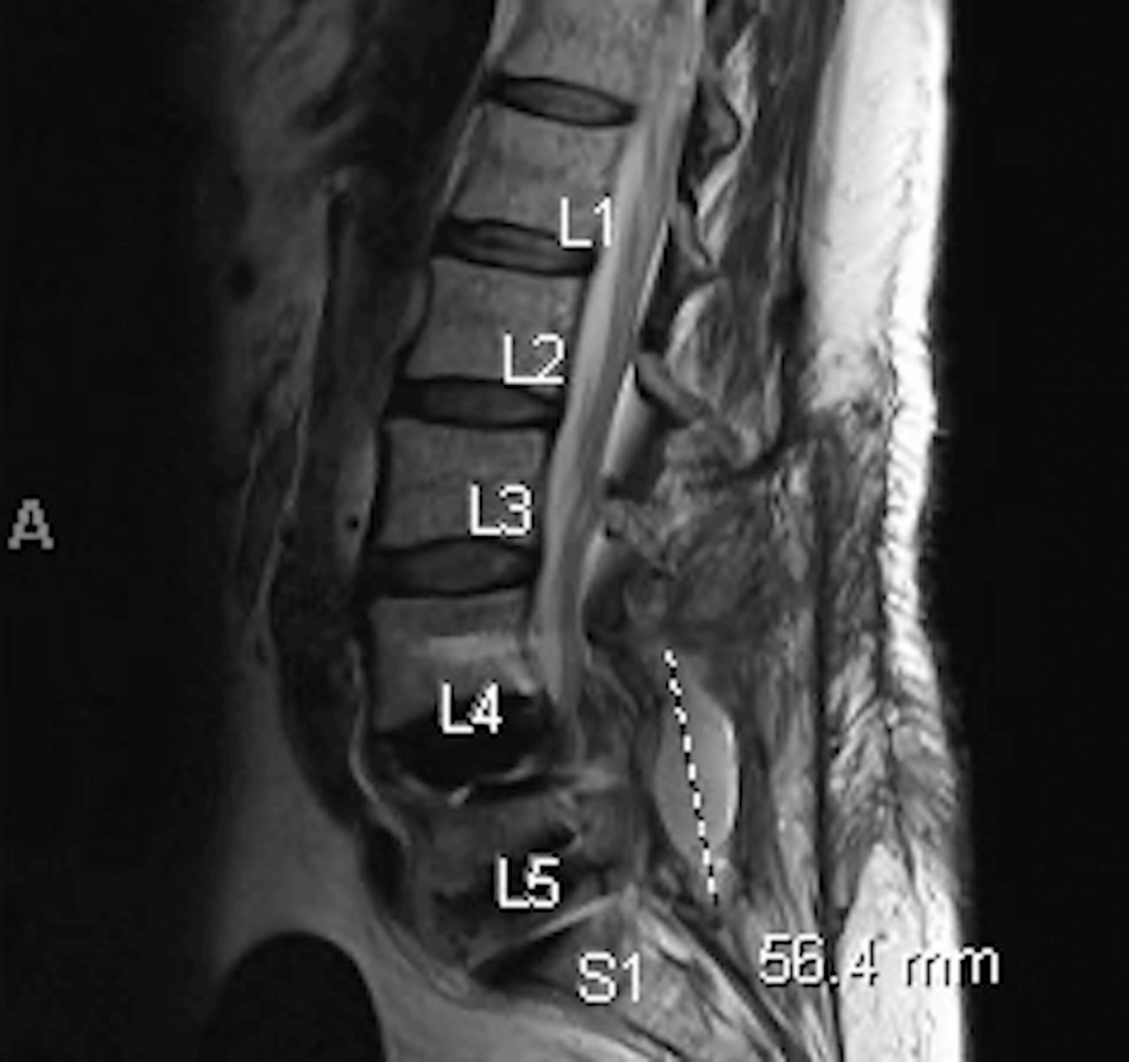
MRI spine of case 3 Fluid collection in proximity to the L5-S1 fusion

A 55-year-old woman with a history of ulcerative colitis, hypothyroidism, depression, tobacco use, chronic obstructive pulmonary disease, hypertension, and hyperlipidemia was evaluated for chronic back pain due to lumbar spine stenosis from spondylolisthesis. She underwent lumbar spinal surgery with allograft transplantation, and two months after the surgery, she developed back pain, weight loss, night sweats, shortness of breath, and cough. During the same period, she was notified that she had received a bone graft contaminated with MTB. IGRA testing was done and returned positive, and she was started on RIPE+ B6 therapy. The patient then underwent an MRI of the spine that showed fluid collection in the L5-S1 fusion and had drainage of the fluid by interventional radiology (Figure 2).

The fluid AFB smear, culture, and MTB-PCR were negative. Her regimen was eventually transitioned to rifampin, isoniazid, moxifloxacin, and pyridoxine, which she tolerated with minimal side effects. 

Case 4 

**Figure 3:**
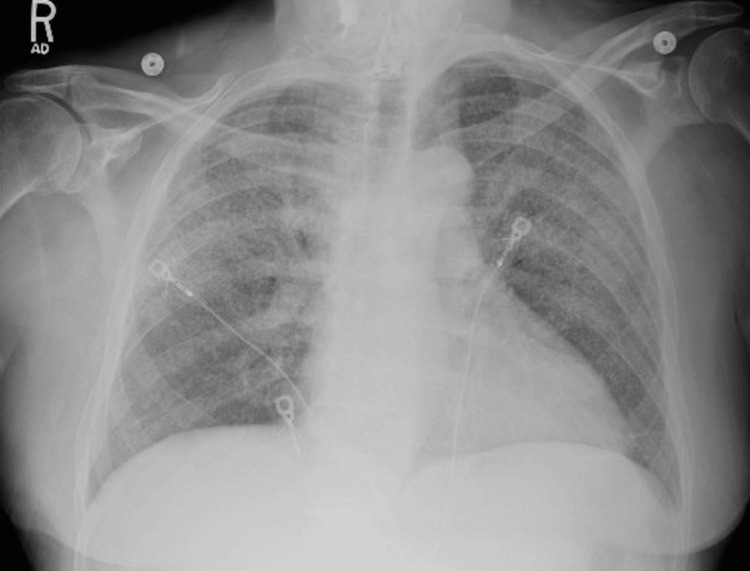
CXR of case 4 CXR showing diffuse hazy opacities in all lung fields

**Figure 4:**
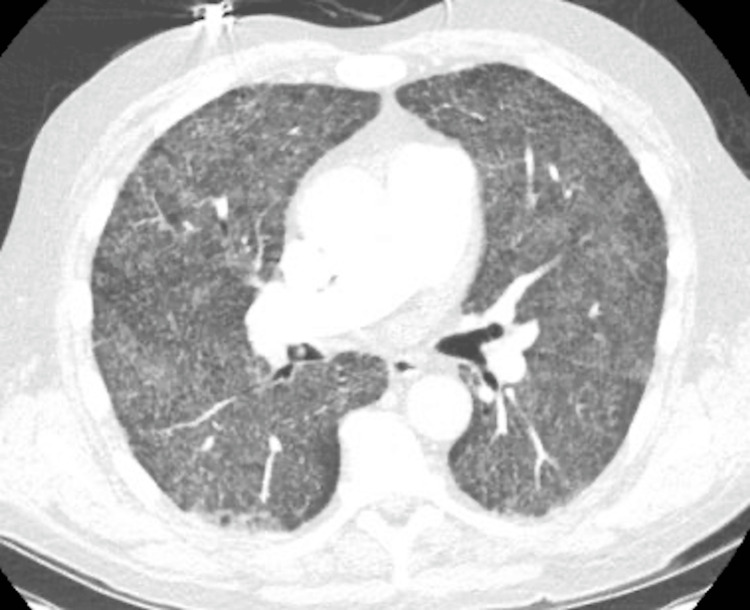
CT chest of case 4 A representative cut from the CT Chest showing diffuse ground-glass opacities and interstitial thickening

A 69-year-old man with a history of hypertension, hyperlipidemia, remote diagnosis of inactive sarcoidosis, former smoking history, GERD, and chronic back pain from spinal stenosis and neurogenic claudication underwent lumbar spine surgery with allograft transplantation. The patient presented three months after the lumbar spine surgery with two weeks of shortness of breath and fatigue. The CT scan of the chest showed diffuse pulmonary interstitial thickening, ground glass opacities, and multiple bilateral pulmonary nodules (Figures 3, 4).

Due to the concern for pulmonary edema, the patient was started on an intravenous diuretic with furosemide without improvement in his symptoms. He underwent bronchoscopy with bronchoalveolar lavage and transbronchial biopsy. After this procedure, the patient was started on systemic steroids due to concern about the possibility of inhalational injury from cleaning product exposure prior to his presentation, given the failure to resolve. The procedure was complicated by pneumothorax, which required temporary small-bore chest tube placement. Due to low suspicion of active tuberculosis at this stage, but with positive IGRA testing, the patient was started on treatment for latent TB and was discharged with plans for outpatient follow-up. After discharge, the patient was notified by the surgical team about exposure to MTB via contaminated bone graft during the spinal surgery. The trans-bronchial biopsy subsequently showed necrotizing granulomas. The MTB-PCR and AFB stains from the bronchoalveolar were negative, but the culture grew MTB complex. The MRI of the spine showed no evidence of fluid collection or inflammation. The patient started RIPE therapy with pyridoxine, and his steroids were weaned off. His sputum cultures cleared after four to five weeks of therapy, and he eventually transitioned to rifampin, isoniazid, moxifloxacin, and pyridoxine, which he continued to tolerate very well with minimal side effects. 

All four patients completed their 12-month medication course for MTB disease and were cleared in July 2024 (Table 1). 

**Table 1:** Characteristics of cases Specific laboratory, imaging, and clinical findings for each patient

	Case 1	Case 2	Case 3	Case 4
Age (years)	70	73	55	69
Sex	Male	Female	Female	Male
Comorbidities	Hypertension, aortic stenosis, prior minimal tobacco use, chronic back pain, and sarcoidosis	Heart failure, aortic stenosis, coronary artery disease, sick sinus syndrome, osteoarthritis, hypertension, diabetes mellitus, chronic kidney disease, paroxysmal atrial fibrillation, hyperlipidemia, and chronic back pain	Ulcerative colitis, hypothyroidism, depression, smoking, chronic obstructive pulmonary disease, hypertension, hyperlipidemia, prediabetes mellitus, and chronic back pain	Hypertension, hyperlipidemia, remote history of sarcoidosis, former smoking history, GERD, and chronic back pain
Indication for spine surgery and bone graft	Chronic back pain due to spondylolisthesis of the lumbar region and severe spinal stenosis	Chronic back pain due to spondylolisthesis of the lumbar region and spinal stenosis	Chronic back pain due to lumbar spine stenosis from spondylolisthesis	Chronic back pain from spinal stenosis and neurogenic claudication
Date of surgery (month/year)	04/2023	05/2023	05/2023	07/2023
Presenting complaints after bone graft surgery	Fever, fatigue, headache, and myalgias	Recurrent lower back pain, intermittent low-grade fever, malaise, and anorexia	Back pain, weight loss, night sweats, shortness of breath, and cough	Shortness of breath and fatigue
Onset of symptoms after bone graft surgery (weeks)	4 weeks	4 weeks	8 weeks	12 weeks
Laboratory findings: CRP (0.0-5.0 mg/L)	84.8 (elevated)	26.6 (elevated)	None	None
ESR (0-20 mm/h)	119 (elevated)	None	None	None
IGRA	Positive	Positive	Positive	Positive
Blood culture	Negative	Negative	Negative	Negative
Tissue/fluid culture and biopsy	None	Negative	Negative	MTB-complex and necrotizing granulomas
Imaging MRI	Soft tissue edema at the laminectomy bed with tiny postsurgical peripherally enhancing fluid collection	None	Fluid collection in the L5-S1 fusion	Evidence of fluid collection or inflammation
CT scan	None	L5 diskitis and osteomyelitis	None	Diffuse pulmonary interstitial thickening, ground glass opacities, and multiple bilateral pulmonary nodules
Type of surgical management	None	Bilateral lumbar incision, drainage, and debridement of the spine infection	Interventional radiology drainage of the fluid	None
Outcome	Treatment complicated by rash and hyperuricemia Treatment modified to rifampin, isoniazid, moxifloxacin, and pyridoxine, completed 12 months total	Treatment complicated by hyperuricemia and gout Treatment modified to rifampin, isoniazid, moxifloxacin, and pyridoxine, completed 12 months total	Treatment complicated by diarrhea and nausea Treatment modified to rifampin, isoniazid, and moxifloxacin, and pyridoxine, completed 12 months total	Tolerated treatment Treatment modified to rifampin, isoniazid, moxifloxacin, and pyridoxine, completed 12 months total

## Discussion

Back pain is one of the most commonly reported medical symptoms, affecting millions of individuals each year. Back pain accounted for about 60% of all patients presenting to the primary care physician [1]. The causes of back pain range from mechanical or structural diseases of the back to inflammatory disease and referred pain from abdominal disease, which can include psychiatric conditions [5,6]. Some of the mechanical or structural diseases causing back pain include degenerative disc disease, herniated or ruptured discs, spondylolisthesis, or spinal stenosis. The mainstay of treatment of back pain is conservative care with rehabilitation, but in patients with severe disease with nerve compression from herniated disc, spondylolisthesis, fracture, or spinal canal stenosis, surgery can be helpful [1,2,6]. 

The bone is well known to have the ability to regenerate completely, and the regeneration of the bone is usually stimulated or augmented by distraction osteogenesis, bone transport, and the use of bone grafts [7]. Bone grafting is a surgical procedure that aids bone regeneration by using the principles of osteoinduction, osteogenesis, and osteoconduction [7]. In osteoinduction, the bone morphogenetic proteins (BMPs) and other growth factors aid the undifferentiated cells to become active osteoblasts, while in osteogenesis, osteoprogenitor cells aid bone growth and remodeling. In osteoconduction, the bone graft acts as a scaffold to guide the reparative growth of the natural bone. 

The autologous bone graft was previously considered the gold standard due to the embedded three properties of osteoinduction, osteogenesis, and osteoconduction, but the acquisition is associated with donor site pain, increased operative time, and insufficient availability [3,4,7,8]. An alternative to autologous bone graft is the use of allograft. Bone allograft is readily available because it can often be obtained from donor cadavers [8]. 

The process of the allogeneic graft harvesting begins with the history of the donor and screening designed to eliminate donors with significant risk for human immunodeficiency virus (HIV), hepatitis B or C, sexually transmitted diseases, systemic viral or bacterial infections, autoimmune diseases, clinically significant metabolic bone disease, malignancy, or systemic toxicity [9]. Patients with unknown cause of death are also excluded from donating [9]. The blood testing required by the AATB and the Food and Drug Administration is intended to identify HIV, hepatitis B virus, syphilis, hepatitis C virus, West Nile virus, human T cell lymphocyte virus I & II, *Chlamydia trachomatis* and *Neisseria gonorrhoeae*, and cytomegalovirus (CMV) [9-11]. After harvesting, the processing of the allograft further includes lyophilization (freeze-drying) or freezing (fresh frozen) to decrease antigenicity and eradicate infectious agents [8]. While the allograft is an alternative to autograft, the allograft lacks vascularization, significant osteoinductive, or osteogenic properties and has slow incorporation into the host bone, and these properties are further impacted by the processing of the allograft. 

The transmission of MTB through tissue transplantation is rare, but in 2021, there was a nationwide tuberculosis outbreak that was linked to contaminated bone allograft with MTB, which affected 113 patients [12,13]. In all, 73% of the patients who were exposed to the contaminated bone allograft developed extrapulmonary TB disease at the surgical site, while 24% had pulmonary disease [12]. The risk of transmission of MTB was not limited to the recipients but also involved some of the health care providers (HCPs). Of the 5985 HCPs that were exposed, 73 of them developed latent TB infection [12]. In 2023, there was the second nationwide tuberculosis outbreak that was due to bone allografts containing *M. tuberculosis*, in which 36 recipients were affected [14]. Unfortunately, thus far, multiple patients have received laboratory-confirmed TB disease diagnoses, including our patients, and two patients have died of MTB infection [14]. 

In our cases, the most common symptoms were low-grade fever, fatigue, malaise, and recurrent back pain. The onset of symptoms varied from four to 12 weeks after bone grafting, but two of the patients developed symptoms four weeks after the surgery. In the patients with recurrent back pain, all had spinal involvement that ranged from soft tissue edema, fluid collection, and diskitis to concern for osteomyelitis. In the two patients with the development of post-operative spinal collections who underwent incision and drainage, the AFB smear and culture were negative. One of the other patients developed pulmonary MTB, and there was a delay in diagnosis due to low suspicion until the team was notified about the patient’s exposure to MTB via bone graft during the spine surgery. All four of our patients had a positive IGRA test. All the patients were started on the standard RIPE therapy with pyridoxine, which was then transitioned to rifampin, isoniazid, and moxifloxacin as per evolving CDC recommendations for improved bone penetration [15]. Our patients reported rash, tendonitis, fatigue, weakness, gout, and nausea. Labs that may have been attributable to TB therapy revealed hyperuricemia and anemia. Most of our patients reported extreme frustration with having contracted TB and having to adhere to a prolonged course of therapy with side effects. In retrospect, the availability of a support group would likely have been very beneficial to each of our patients. 

In response to both outbreaks, the AATB has recently published new guidelines, which include recommended criteria and a literature review to aid with screening out cases that may have MTB [16]. These include recommending against utilizing TB skin-testing (TST) or IGRA to rule out donors potentially infected with MTB and instead utilizing culture-based testing, with a full eight weeks of laboratory observation to declare negative results. In addition, donors with sepsis at the time of death should be excluded from donation. Chronically ill individuals may be at higher risk for harboring MTB, and the working group recommends caution when accepting donations in these situations. Other criteria for ineligibility to donate include age >65, incarceration <2 years prior, exposure to active TB <2 years prior, history of latent tuberculosis infection (LTBI), and relevant high-risk travel and immigration history. 

After the first outbreak in 2021, the tissue procurement organizations implemented the use of nucleic acid amplification testing for MTB in the bone allografts, but this is not the most sensitive test available. This test did not detect the MTB in the tissue that was implicated in the second outbreak, and liquid cultures took 40 days after inoculation for MTB to be identified, which is longer than the typical 14- to 21-day period [14]. To address this preventable recurrence, a viewpoint in JAMA in May 2023 argued to formalize an FDA requirement that bone matrix companies adhere to a strict mandatory screening process instead of an optional set of recommendations [17].

## Conclusions

After tissue transplantation, especially allogeneic bone graft, clinicians should maintain a higher index of suspicion for MTB infection in patients presenting with low-grade fever, fatigue, malaise, and recurrent back pain or respiratory symptoms, along with a workup for more common causes of post-operative infection. In patients with manifestations limited to the spine, while AFB smear and culture might be negative, as seen in our cases and previously reported cases, the use of IGRAs can help to raise an index of suspicion and guide initiation of treatment. 

Given the significant morbidity and mortality associated with grafting MTB-contaminated bone, we recommend increased caution during donor procurement and support steps to improve standards of screening and exclusion, including a full eight weeks of allowance for cultures to result prior to grafting, as stipulated in the AATB guidelines referenced above. 

In addition to these standards, we support the viewpoint in JAMA in May 2023 to formalize an FDA requirement that bone matrix companies adhere to a strict mandatory screening process instead of an optional set of recommendations. 
